# 1023. Suppressed Switch to Bictegravir/Emtricitabine/Tenofovir Alafenamide vs. Dolutegravir/Lamivudine: Virologic Failure and Durability

**DOI:** 10.1093/ofid/ofad500.054

**Published:** 2023-11-27

**Authors:** Gerald Pierone, Jennifer S Fusco, Laurence Brunet, Michael Sension, Megan Dunbar, Joshua Gruber, Douglas Dieterich, Gregory P Fusco

**Affiliations:** Whole Family Health Center, Vero Beach, FL; Epividian, Inc., Durham, North Carolina; Epividian, Inc., Durham, North Carolina; CAN Community Health, Fort Lauderdale, Florida; Gilead Sciences, Forest City, California; Gilead Sciences, Forest City, California; Mount Sinai Healthcare System, New York, New York; Epividian, Inc., Durham, North Carolina

## Abstract

**Background:**

In the US, two common single-tablet regimens for HIV treatment are bictegravir/emtricitabine/tenofovir alafenamide (B/F/TAF) and dolutegravir/lamivudine (DTG/3TC). We sought to compare B/F/TAF and DTG/3TC in virologically suppressed, treatment-experienced people with HIV in the OPERA^®^ cohort.

**Methods:**

All treatment-experienced adults with HIV switching to B/F/TAF or DTG/3TC (01Aug2020-30Jun2022) with a viral load (VL) < 200 copies/mL at switch and ≥1 follow-up VL were included. Confirmed virologic failure (VF) was defined as 2 consecutive VL ≥200 copies/mL or regimen discontinuation following a VL ≥200 copies/mL; VL ≥50 copies/mL was used in a sensitivity analysis. Discontinuation was defined as any regimen modification or a treatment gap >45 days. Incidence rates (Poisson regression) and hazard ratios (Cox proportional hazard models) were estimated with inverse probability of treatment weights (IPTW) to adjust for race, payer, CD4 count and eGFR at baseline. Covariate balance was assessed with standardized mean differences; values ≤0.10 indicated adequate balance.

**Results:**

On B/F/TAF, 3713 individuals were followed for a median of 16 months (interquartile range: 11, 22). On DTG/3TC, 2327 individuals were followed for a median of 15 months (10, 21). The distribution of key characteristics differed between groups; balance was achieved with IPTW (Table 1). VF_≥200_ incidence rates per 100 person years were low (B/F/TAF: 1.7; DTG/3TC: 2.1); risk with B/F/TAF was not statistically different than with DTG/3TC (HR_≥200_: 0.84 [95% CI: 0.59, 1.18]). VF_≥50_ incidence rates were higher, but risk did not differ between groups (HR_≥50_: 1.04 [0.86, 1.26]; Fig 1). All-cause regimen discontinuation was less likely with B/F/TAF than DTG/3TC (HR: 0.83; 95% CI: 0.73, 0.94; Fig 2). Treatment-related discontinuation (i.e., last VL ≥200 copies/mL, adverse diagnosis, side effect, lab abnormality) was identified in 6% of B/F/TAF and 9% of DTG/3TC discontinuers.

**Figure:**
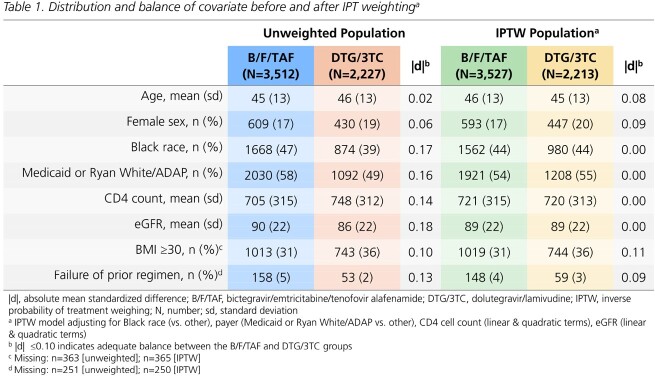

**Figure:**
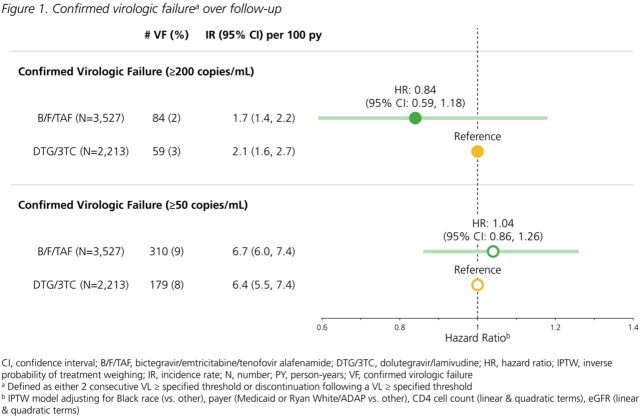

**Figure:**
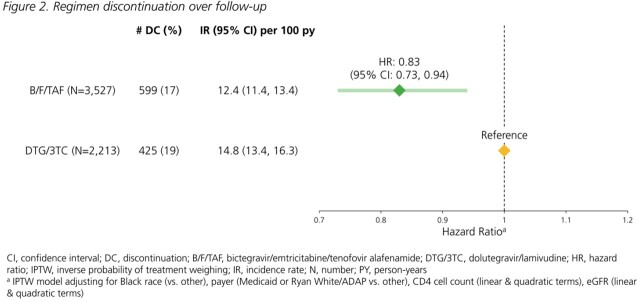

**Conclusion:**

In this real-world US cohort, virologically suppressed individuals switching to B/F/TAF were less likely to discontinue their regimen than those switching to DTG/3TC. VF was infrequent and no statistical difference was observed in the risk of VF between regimens over the study duration.

**Disclosures:**

**Jennifer S. Fusco, BS**, Epividian, Inc.: Salary|Epividian, Inc.: Ownership Interest|Epividian, Inc.: Stocks/Bonds **Laurence Brunet, PhD**, Epividian, Inc.: Salary|Epividian, Inc.: Stocks/Bonds **Michael Sension, MD**, Gilead: Advisor/Consultant|Gilead: Honoraria|Viiv: Advisor/Consultant|Viiv: Grant/Research Support|Viiv: Honoraria **Megan Dunbar, PhD**, Gilead: Employment **Joshua Gruber, PhD**, Gilead Sciences, Inc: Employee|Gilead Sciences, Inc: Stocks/Bonds **Douglas Dieterich, MD**, Abbvie: Advisor/Consultant|Abbvie: Honoraria|Gilead: Advisor/Consultant|Gilead: Honoraria|Merck: Advisor/Consultant|Merck: Honoraria **Gregory P. Fusco, MD, MPH**, Epividian, Inc.: Board Member|Epividian, Inc.: Ownership Interest|Epividian, Inc.: Stocks/Bonds

